# Supplementary material for: Development of Machine Learning Models for Prediction of Smoking Cessation Outcome
Source: Int J Environ Res Public Health. 2021 Mar 5;18(5):2584. doi: 10.3390/ijerph18052584 (PMC7967540; doi:10.3390/ijerph18052584)
Supplement: Supplementary file 1 [file ijerph-18-02584-s001.pdf]

Supplementary files

Table S1. Homogeneity testing between the training and test groups.

Categorical variables	χ^2 value	*p*
Sex	0.2892	0.5907
Ambition	1.7924	0.1806
Physician clinics visit	0.7391	0.3899
Educator clinics visit	0.9227	0.3368
Therapy	0.1168	0.7325
**Continuous variables**	**F value**	***p***
Age (years)	1.04	0.5076
Body weight (kg)	1.10	0.1567
Exhaled CO levels (ppm)	1.04	0.5102
Number of cigarettes smoked per day at baseline (stick)	1.09	0.2046
Duration of smoking (years)	1.01	0.8982
FTND 1	1.01	0.9465
FTND 2	1.02	0.7583
FTND 3	1.00	0.9586
FTND 4	1.02	0.6997
FTND 5	1.09	0.1888
FTND 6	1.02	0.7223
FTND score (point)	1.08	0.2665

Table S2. Pearson's correlation coefficient matrix of predictors.

	Age	Sex	Weight	Ambition	CO level	Therapy	Cig per d	Duration	FTND 1	FTND 2	FTND 3	FTND 4	FTND 5	FTND 6	FTND score	Doc OPD	Edu OPD
Age	1.00000	0.06268*	-0.10977*	-0.00119	-0.11335*	-0.01639	0.02727	0.83704*	0.00727	-0.01718	-0.13481*	-0.05886*	-0.00033	0.05997*	-0.02907*	-0.05132*	0.12280*
Sex	0.06268*	1.00000	0.43658*	0.00130	0.03895*	0.03541*	0.05312*	0.16036*	0.08190*	-0.04099*	-0.04654*	-0.00576	-0.08166*	-0.00282	-0.01779	-0.02319	0.03876*
Weight	-0.10977*	0.43658*	1.00000	0.00988	0.02796	0.04078*	0.06782*	-0.04123*	0.10228*	-0.00855	0.01103	-0.00713	-0.02225	0.01193	0.03437*	0.00842	0.01988
Ambition	-0.00119	0.00130	0.00988	1.00000	0.04031*	0.17458*	0.07031*	0.00372	0.04363*	0.06031*	0.05614*	0.06467*	0.05399*	0.06088*	0.08834*	0.24484*	-0.17632*
CO level	-0.11335*	0.03895*	0.02796	0.04031*	1.00000	0.03341*	0.37728*	-0.06504*	0.28149*	0.12030*	0.08797*	0.05514*	0.19111*	0.04846*	0.24952*	0.00471	0.06284*
Therapy	-0.01639	0.03541*	0.04078*	0.17458*	0.03341*	1.00000	0.06402*	-0.00755	0.04846*	0.03491*	0.04356*	0.03756*	0.02631	0.06490*	0.06727*	0.48466*	-0.19362*
Cig per d	0.02727	0.05312*	0.06782*	0.07031*	0.37728*	0.06402*	1.00000	0.09285*	0.60489*	0.11548*	0.14855*	0.15917*	0.24261*	0.10052	0.44337*	0.12384*	-0.04805*
Duration	0.83704*	0.16036*	-0.04123*	0.00372	-0.06504*	-0.00755	0.09285*	1.00000	0.08656*	0.01430	-0.04858*	0.00799	0.05679*	0.06794*	0.06461*	-0.04772*	0.11810*
FTND 1	0.00727	0.08190*	0.10228*	0.04363*	0.28149*	0.04846*	0.60489	0.08656	1.00000	0.13619*	0.19526*	0.22510*	0.28855*	0.12494*	0.65227*	0.06194*	-0.08936*
FTND 2	-0.01718	-0.04099*	-0.00855	0.06031*	0.12030*	0.03491*	0.11548*	0.01430	0.13619*	1.00000	0.22084*	0.24702*	0.40345*	0.32974*	0.57624*	0.09246*	-0.05805*
FTND 3	-0.13481*	-0.04654*	0.01103	0.05614*	0.08797*	0.04356*	0.14855*	-0.04858*	0.19526*	0.22084*	1.00000	0.34102*	0.26020*	0.26215*	0.56110*	0.11409*	-0.10862*
FTND 4	-0.05886*	-0.00576	-0.00713	0.06467*	0.05514*	0.03756*	0.15917*	0.00799	0.22510*	0.24702*	0.34102*	1.00000	0.23171*	0.23121*	0.55785*	0.08809*	-0.11871*
FTND 5	-0.00033	-0.08166*	-0.02225	0.05399*	0.19111*	0.02631	0.24261*	0.05679*	0.28855*	0.40345*	0.26020*	0.23171*	1.00000	0.25183*	0.72978*	0.05924*	-0.05011*
FTND 6	0.05997*	-0.00282	0.01193	0.06088*	0.04846*	0.06490*	0.10052*	0.06794*	0.12494*	0.32974*	0.26215*	0.23121*	0.25183*	1.00000	0.52738*	0.18663*	-0.11567*
FTND score	-0.02907*	-0.01779	0.03437*	0.08834*	0.24952*	0.06727*	0.44337*	0.06461*	0.65227*	0.57624*	0.56110*	0.55785*	0.72978*	0.52738*	1.00000	0.14807*	-0.13889*
Doc OPD	-0.05132*	-0.02319	0.00842	0.24484*	0.00471	0.48466*	0.12384*	-0.04772*	0.06194*	0.09246*	0.11409*	0.08809*	0.05924*	0.18663*	0.14807*	1.00000	-0.51515*
Edu OPD	0.12280*	0.03876*	0.01988	-0.17632*	0.06284*	-0.19362*	-0.04805*	0.11810*	-0.08936*	-0.05805*	-0.10862*	-0.11871*	-0.05011*	-0.11567*	-0.13889	-0.51515*	1.00000

*, $p<0.05$.
